# A review of research-supported group treatments for drug use disorders

**DOI:** 10.1186/s13011-021-00371-0

**Published:** 2021-06-21

**Authors:** Gabriela López, Lindsay M. Orchowski, Madhavi K. Reddy, Jessica Nargiso, Jennifer E. Johnson

**Affiliations:** 1 Center for Alcohol and Addiction Studies, Brown University, Providence, RI 02912 USA; 2 Alpert Medical School of Brown University, Department of Psychiatry and Human Behavior, Providence, RI 02904 USA; 3 Walter Reed Army Institute of Research, Silver Spring, MD 20910 USA; 4 Massachusetts General Hospital, Harvard Medical School, Boston, MA 02115 USA; 5 Division of Public Health, Michigan State University, Flint, MI 48502 USA

**Keywords:** Treatment, Substance use disorders, Group, Group therapy, Review

## Abstract

This paper reviews methodologically rigorous studies examining group treatments for interview-diagnosed drug use disorders. A total of 50 studies reporting on the efficacy of group drug use disorder treatments for adults met inclusion criteria. Studies examining group treatment for cocaine, methamphetamine, marijuana, opioid, mixed substance, and substance use disorder with co-occurring psychiatric conditions are discussed. The current review showed that cognitive behavioral therapy (CBT) group therapy and contingency management (CM) groups appear to be more effective at reducing cocaine use than treatment as usual (TAU) groups. CM also appeared to be effective at reducing methamphetamine use relative to standard group treatment. Relapse prevention support groups, motivational interviewing, and social support groups were all effective at reducing marijuana use relative to a delayed treatment control. Group therapy or group CBT plus pharmacotherapy are more effective at decreasing opioid use than pharmacotherapy alone. An HIV harm reduction program has also been shown to be effective for reducing illicit opioid use. Effective treatments for mixed substance use disorder include group CBT, CM, and women’s recovery group. Behavioral skills group, group behavioral therapy plus CM, Seeking Safety, Dialectical behavior therapy groups, and CM were more effective at decreasing substance use and psychiatric symptoms relative to TAU, but group psychoeducation and group CBT were not. Given how often group formats are utilized to treat drug use disorders, the present review underscores the need to understand the extent to which evidence-based group therapies for drug use disorders are applied in treatment settings.

## Background

Drug use disorders are a significant public health concern in the United States. According to the National Epidemiologic Survey of Alcohol and Related Conditions-III, the lifetime prevalence rate of DSM-5 drug use disorders is 9.9%, which includes amphetamine, cannabis, club drug, cocaine, hallucinogen, heroin, opioid, sedative/tranquilizer, and solvent/inhalant use disorders [1]. Drug use disorders are defined in terms of eleven criteria including physiological, behavioral and cognitive symptoms, as well as consequences of criteria, any two of which qualify for a diagnosis [2, 3]. The individual and community costs of drug use are estimated at over $193 billion [4, 5] and approximately $78.5 billion [6] for opioids alone. Consequences include overdose [7], mental health problems [8], and a range of medical consequences such as human immunodeficiency virus [9, 10], hepatitis C virus [9], and other viral and bacterial infections [11].

Evidence-based practice was formally defined by Sackett et al. [12] in 1996 to refer to the “conscientious, explicit, and judicious use of current best evidence in making decisions about the care of individual patients” (p. 71). In 2006, the American Psychological Association [13] developed a policy on evidence-based practice (EBP) of psychotherapy, which emphasized the integration of best research evidence (i.e., data from meta-analyses, randomized controlled trials, effectiveness trials, and other forms of systematic case studies and reviews) with clinical expertise and judgment to deliver treatment in the context of a patient’s individual needs, preferences and culture. The shift towards EBP for substance use disorders has multiple benefits for practitioners and patients, including an increased focus on the implementation of treatments that are safe and cost-effective [14]. A recent survey of clinicians’ practices with substance use treatment found that clinicians often conducted therapy in groups [15]. While most clinicians who completed the survey reported use of evidence-based treatment practices (EBT) some also reported the use of non-EBT practices [15]. Ensuring that clinicians can readily access information regarding the current state of evidence regarding group-based therapies for substance use disorders is critical for fostering increased use of EBTs.

Although any effort to summarize a literature as large and complex as the psychological treatment literature is useful, there are several limitations. With few exceptions, research-supported treatment lists categorize treatments by formal change theory (e.g., cognitive-behavioral, interpersonal) and describe little about the context, format, or setting in which treatments were conducted and tested [16]. As a result, it is often difficult to ascertain from existing resources whether research supported treatments were conducted in group or individual format. A group format is often used in substance use treatment [17] and aftercare programs [18–22]**.** The discrepancy between the wide-spread use of group therapy in clinical practice and the relative paucity of research on the efficacy of group treatments has been noted by treatment researchers [23] and clinicians [24]. According to Lundahl’s [25] 2010 meta-analysis of studies evaluating the efficacy of motivational interviewing (MI), a commonly used treatment for substance use disorders, examination of the 119 studies concluded that studies of MI in a group format were too rare to draw solid conclusions about the efficacy of group MI. Also, it is possible that efficacy of treatments developed for individual delivery will be altered when delivered in a group format and vice versa. Given the limited empirical inquiry on group treatments for substance use, a framework organizing the literature on the efficacy of group therapy to treat substance use disorders would be useful. There is also a need for a more recent rigorous review of the empirical evidence to support group-based treatments for substance use disorders. Over 15 years ago, Weiss and colleagues conducted a review of 24 treatment outcome studies within the substance use disorder intervention literature comparing group therapy to other treatments conditions (i.e., no group therapy, individual therapy, group therapy plus individual therapy), and found no differences between group and individual therapy [26].

Given the importance of understanding the current evidence base for group-delivered treatments for substance use disorders, the present review sought to provide a summary of the literature on the benefits of group treatments for drug use disorders. Group treatments are potentially cost-effective, widely disseminable, and adaptable to a variety of populations but are lagging individual treatments in terms of research attention. Thus, highlighting characteristics of group treatments that are potentially efficacious is of import to stimulate further empirical inquiry. The review is organized by drug type (cocaine, methamphetamine, marijuana, opiate, mixed substance use disorders; SUD) and co-occurring SUD and psychiatric problems. We excluded studies focused on alcohol use disorder alone as this literature is summarized elsewhere (see Orchowski & Johnson, 2012). Given research suggesting that several factors impact outcomes of group treatments, including formal change theory driving the treatment approach (i.e., cognitive-behavioral, motivational interviewing), as well as patient factors [27], the review begins by first reviewing each theory of change (i.e., type of treatment), and then concludes by summarizing the research examining the extent to which patient factors influence the efficacy of group treatments for SUD.

## Method

**Fig. 1:**
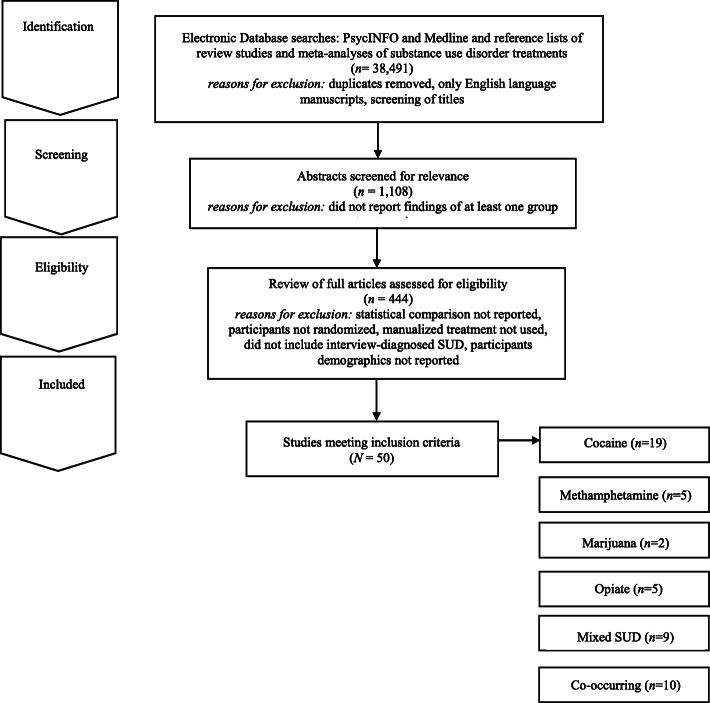
Electronic Search Strategy Flowchart

To locate studies that evaluated a group treatment for SUD that met review inclusion criteria, the authors conducted a comprehensive literature search of PsycINFO and MedLine through 2020. Three individuals then examined abstracts of the articles for relevance. In addition, the authors utilized the reference lists of review studies and meta-analyses of SUD- treatments to locate additional studies that might meet the review inclusion criteria. The authors and a research assistant then reviewed full articles with relevance to the current study and excluded any studies that did not meet the review inclusion criteria (see Fig. 1).

For inclusion in the review, studies needed to meet the following criteria: 1) report the findings of at least one group treatment; 2) provide at least one statistical comparison between the group treatment and a control condition; 3) randomize participants between the group treatment and control condition; 4) utilize a manualized treatment; 5) include patients with an interview-diagnosed SUD; and 6) provide information regarding the demographic characteristics of the participants in the study. Studies’ methods and results were used for data extraction. Studies which maintained a primary focus on the treatment of SUD, but also included treatment of a co-occurring psychiatric condition, were included in the review. Studies which included alcohol use as a comorbid diagnosis along another substance use were included. Studies examining the efficacy of group treatment for only alcohol use were excluded. The final set of articles included were 50 research studies that utilized a group treatment modality for the treatment of SUD, including separately examining cocaine, methamphetamine, marijuana, opioid, mixed substance, or SUD with comorbid psychiatric problems in adults.

It should be noted that several studies that met inclusion criteria were not reported in the present review because they did not report the use of a specific screening instrument for SUD as a part of the study inclusion/exclusion criteria. These studies are as follows and include these comparisons: group-based relational therapy [28] two studies by Guydish et al. [29, 30] comparing a day treatment program to residential treatment (RT) program, a day treatment program to a coping skills group [31], standard care to a harm reduction group [32], 12 step group to a CBT group [33], medical management treatment (MMT) with CBT group to an MMT plus treatment reinforcement plan [34], treatment as usual to contingency management (CM) [35], professionally led recovery training group to treatment as usual (TAU) [36], two 4 month residential treatment programs [37], varying lengths of therapeutic community program (TPC) with and without relapse prevention [38], and Information and Referral plus peer advocacy to a Motivational group with CBT group [39].

### Review of evidence-based theories of change

The 50 research studies meeting inclusion criteria tested the following group treatment modalities: contingency management (CM), motivational interviewing (MI), relapse prevention (RP), social support (SS), cognitive-behavioral (CBT), coping skills (CS), harm reduction (HR), cognitive therapy (CT), drug counseling (DC), recovery training (RT), standard group therapy (SGT), family therapy (FT), intensive group therapy (IGT), 12 step facilitation group therapy (12SG), relational psychotherapy mothers’ group (RPMG), psychoeducational therapy group (PET), behavioral skills (BS), and seeking safety (SS). Below, we briefly review the theory of change that drives each of these treatments.

Several treatment approaches are grounded in behavioral therapies and/or cognitive therapies. Broadly, cognitive therapy is an approach that focuses exclusively on targeting thoughts that are identified as part of a diagnosis or behavioral problem [28]. Cognitive-behavioral (CBT) therapy is an approach that targets specific symptoms, thoughts, and behaviors that are identified as part of a diagnosis or presenting problem [28]. Under the umbrella of CBT several other treatment modalities exist. For example, relapse prevention is a CBT treatment that hypothesizes that there are cognitive, behavioral, and affective mechanism that underlie the process of relapse [40]. Recovery training is a more specific form of relapse prevention, including education on addiction and recovery and reinforcing relapse prevention skills (e.g., understanding triggers, coping with cravings etc.) [41, 42]. Other treatments focus on coping skills more broadly. For example, coping skills treatments include a focus on components of adaptability in interpersonal relationships, thinking and feeling, as well as approaches to self and life [28]. Some treatment approaches also recognize that individuals may not be ready to change their substance use. For example, motivational interviewing is often described as a therapy guiding technique in which the therapist is a helper in the behavior change process and expressed acceptance of the patient [43]. Standard group therapy includes 90 min sessions approximately twice a week in a group setting, [44] whereas intensive group therapy is a heavier dose of standard group therapy that includes 120-min sessions up to five times a week [44]. Psychoeducational therapy group focused on providing information on the immediate and delayed problems of substance use disorders to patients [45]. Lastly, dialectical behavior therapy (DBT) is a type of CBT therapy that focuses on helping regulate intense emotional states and provides skills to reduce arousal levels, and increase mindfulness, emotional regulation, and interpersonal skills [46].

Grounded within behavioral therapies, are behavioral skills training which focused on developing behaviors that are adaptive [28]. Contingency management is a type of behavioral therapy in which patients are reinforced or rewarded for positive behavioral change [47]. Harm reduction is a term for interventions aiming to reduce the problematic effects of behaviors [48]. Several treatment approaches also focus on interpersonal networks and building interpersonal skills. For example, social support is any psychological resources provided by a social network to help patients cope with stress [49]. Twelve-step facilitation group therapy is a more specific form of social support, which focuses on introducing patients to the 12 steps of alcoholics anonymous or related groups (i.e., cocaine or narcotics anonymous) to encourage 12-step meeting attendance in their community [33, 50]. Seeking Safety is a present-focused and empowerment-based intervention focused on coping skills that emphasizes the importance of safety within interpersonal relationships [51]. Drug counseling describes treatment that aims to facilitate abstinence, encourage mutual support, and provide coping skills [52]. Finally, family therapy is a family-based intervention that aims to change, parenting behaviors and family interactions [53]. Overall, there are many overlapping components and skill sets in the models discussed above (See Table 1).

**Table 1:** Therapy subtypes organized by theories of change

Cognitive Behavioral Therapy	Behavioral Therapy	Interpersonal Skills Therapy
Relapse Prevention	Behavioral skills	Social Support
Recovery Training	Contingency Management	Twelve Step Group Therapy
Coping Skills	Harm Reduction	Seeking Safety
Motivational Interviewing		Family Therapy
Standard Group Therapy		Drug Counseling
Psychoeducational Therapy		
Dialectical Behavior Therapy		

### Group-based cocaine use treatments for adults

Nineteen studies were identified that targeted cocaine use and utilized some form of group therapy, the most of any drug in this review (see Table 2). Overall, the studies showed that all of the group therapy modalities included in this review generally reduced cocaine use when compared to treatment as usual (TAU), including day hospital groups [54]. Two studies, Magura et al. (1994) and Magura et al. (2002) did not find group differences between 8 months CBT and 8 months of TAU that consisted of methadone maintenance therapy among 141 patients with cocaine disorder [60, 69]. When compared directly, individuals in CBT groups achieved longer abstinence than individuals in 12 step facilitation groups [33] or low intensity groups [64, 65]. However, in another study, individuals with cocaine dependence receiving 12-step based Group Drug Counseling (GDC; similar to 12-step facilitation) had similar cocaine abstinence outcomes with or without additional individual CBT [41]. This may suggest that group 12-step facilitation is an effective intervention for cocaine dependence. Two studies demonstrated the superiority of CM groups for reducing cocaine use as compared to CBT [62] or TAU groups [61, 62] at 12 weeks [54], 17 weeks [53], 26 weeks [53] and 52 weeks follow up [51]. Therefore, CBT group therapy and contingency management groups appear to be more effective at reducing cocaine use than TAU groups.

**Table 2:** Summary of evidence-based cocaine use group treatment for adults

Study	Patient Characteristics^**a**^	Treatment Description	Manual	Study Characteristics^**b,c**^	Results
Coviello et al., 2001 [54]	94 veterans with CD (DIS) and without psychiatric or medical instability. Mean age 40 years, 0% female, 92% African American.	4-weeks: 1. 12-h per weekday hospital program (DH12; 12-h abbreviated version of a 27-h a weekday hospital program, with 7 h of group treatment, 3 h of educational therapy and 2 h of counseling and case management over five weekdays). 2. 6-h per week outpatient program (OP6; 6-h of groups, 1 h of educational therapy and 1 h of counseling and case management over three weekdays).	1. Yes 2. Yes	RAAT; 4- and 7-month f/u. 93% f/u rate; 39% tx completion.	Across groups, patients reported a 52% reduction in days of cocaine use and experienced significant improvements in employment and psychiatric functioning at 7-month f/u. No difference between DH12 and OP6 programs in terms of abstinence during treatment, treatment completion, treatment or aftercare attendance or any Addiction Severity Index (ASI)-related variable at 4- and 7-month f/u.
Crits-Christoph et al., 1999 [41]; Crits-Christoph et al., 2001 [55]; Siqueland et al., 2002 [56]	487 solicited with CD (Anxiety Disorders Interview Schedule). Mean age 34 years, 23% female, 40% African American.	24 weekly group sessions for 90 min, 36 individual sessions for 50 min, plus 3 monthly booster sessions: 1. Manual guided group drug counseling (GDC; [57]) 2. GDC plus individual supportive-expressive therapy (GDC + SE; [58]), 3. GDC plus individual cognitive therapy (GDC + CT) 4. GDC plus 12-step individual drug counseling (GDC + IDC)	1. Yes 2. Yes 3. Yes 4. Yes	RAAT; 3-, 6-, 9-, and 12-month f/u. 100% f/u rate. 31% tx completion.	IDC + GDC reduced drug composite score more than other treatments over 9- and 12-month f/u. No differences revealed between GDC or GDC + SE or GDC + CT. Superiority of IDC + GDC vs. others did not extend to other addiction associated problems. IDC + GDC stayed in treatment for fewer days than others but were more likely to be abstinent after dropout. Younger, African American, and unemployed patients were retained in treatment for fewer days than others. Higher psychiatric severity kept men in treatment longer but increased women’s risk for drop out. Higher psychiatric severity increased risk for continuing to use drugs after dropout.
Epstein et al., 2003 [59]	193 methadone-maintained outpatients using cocaine (DIS; diagnoses of heroin or CD not required) without psychosis, bipolar, or major depressive disorders, AD, or sedative dependence, medical conditions, pregnancy, cognitive impairment, or and urologic conditions that would preclude urine collection. Mean age of 29 years, 43% female, 70% African American.	Daily methadone and weekly individual counseling for 29 weeks, with baseline treatment (5 weeks), intervention (12 weeks), and maintenance therapy (12 weeks): 1. Voucher condition (CM; contingent on cocaine-negative urine or noncontingent). 2 CM plus CBT based group therapy (CM-CBT; 1x week, 90 min for 12 weeks) 3. Cognitive behavioral group (CBT; 1x week, 90 min for 12 weeks) 4. Control condition (Control; Social support group, 1x week, 90 min for 12 weeks)	1. Yes 2. Yes 2. No (Control; but Yes, for CM) 4. No	RAWC; 12- week f/u. 63% of control completed f/u, 62% of CM completed f/u, 58% of CBT completed f/u, and 57% of CM-CBT completed f/u. 76% of control completed tx, 81% of CM completed tx, 79% of CBT completed tx, and 69% of CM-CBT completed tx.	During treatment, initial effects of CM were dampened by CBT. Posttreatment CM-CBT evidenced positive results compared to others over 12-month f/u. CBT participants were also more likely to acknowledge cocaine use and its effects and to report employment.
Hoffman et al., 1996 [44]	184 referred individuals with cocaine abuse/CD (CIDI and DIS), without dependency on other drugs, or psychosis. Mean age 32 years, 40% female, 95% African American.	4-months with up to 4 vocational assessment/therapy sessions on an individual basis, and up to 4 family group therapy sessions once a month): 1. Standard Group Therapy (SGT; 90-min, 2 sessions per week) with Individual Therapy (IT; 60 min, 2 sessions per week starting month 1 and 1 session thereafter) (SGT + IT) 2. SGT + IT plus Family Therapy (FT; 90-min sessions, 1 session per week starting in month 2) (SGT + IT+FT) 3. SGT only 4. Intensive Group Therapy (IGT; 120-min, 5 sessions per week) with IT (IGT + IT) 5. IGT + IT+FT 6. IGT only	1. Yes 2. Yes 3. Yes 4. Yes 5. Yes 6. Yes 7. Yes	RAAT; 12-month f/u, 66% 12-month f/u rate. Tx completion rates were: 19.1% SGT only, 38.5% SGT + IT, 46.8% SGT + IT+FT, 45.2% IGT only, 34.3% IGT + IT, and 38.5% IGT + IT+FT.	Across groups, patients evidenced significant pre-post treatment gains: reduced regular cocaine use, reduced other drug use, reduced regular alcohol use, and reduced involvement in illegal activities. Regular cocaine users over 12-month f/u were more likely to be female, less educated, have been using cocaine prior to treatment, spent fewer days incarcerated during 12-months post treatment, and have attended fewer treatment sessions.
McKay et al., 1997 [21]; McKay et al., 1999 [20]	132 veterans referred from intensive outpatient treatment with CD (SCID). Mean age 40 years, 0% female, 85% African American.	2 sessions per week for 5 months: 1. Standard aftercare group (ST; addiction counseling and 12-step based) 2. Individual relapse prevention (RP; 1 weekly ST group plus 1 session individual therapy, self-efficacy focused)	1. No 2. Yes	RAAT. 2-yr. f/u, 92% follow-up rate, 43% tx completion.	Complete abstinence rates favored ST but RP was more effective in limiting extent of cocaine use. Self-efficacy predicted cocaine use. Patients reporting commitment to absolute abstinence had better cocaine use outcomes in RP compared to ST. Patients reporting less stringent abstinence goals had better cocaine use outcomes in ST compared to RT. Patients with CD or AD upon entering tx who received RP had better cocaine use outcomes in Months 1–6 and better alcohol use outcomes in Months 13–24 than those in ST. At 2 years, medical outcomes were significantly better for RT compared to ST.
Magura et al., 1994 [60]; Magura, et al., 200,256)	141 patients in methadone maintenance treatment with CD (SCID). Mean age 39 years, 33% female, 26.2% African American, 39.7% Hispanic.	8 months of treatment: 1. Cognitive behavioral therapy plus treatment reinforcement plan (CBT; Matrix model; In Phase I, subjects participated in a 4-month CBT program with two individual and three group sessions per week. Phase II consisted of two group sessions per week. 2. Treatment as usual (TAU; standard methadone maintenance therapy)	1. Yes 2. No	RAWC across tx site (2 sites offered CBT, 2 sites TAU); 4- and 12-month f/u. 76% 12-month f/u rate. For CBT 56% tx completion for Phase I and 51% tx completion for Phase II.	Cocaine use declined significantly from baseline to 4- and 12-month f/u across groups. CBT participants rated the quality of their counseling relationship higher and obtained more supportive services than TAU. Group was not associated with outcome. Measures associated with poorer outcomes across both groups were: currently enrolled in methadone treatment, higher cocaine use frequency, greater cocaine use associated problem recognition, and an ambivalence toward methadone treatment.
Petry et al., 2007 [61]	387 patients in intensive outpatient with cocaine abuse or CD (SCID) and without psychosis, suicidal, or pathological gambling. Mean age 36 years, 50% female, 51% African American.	12-weeks: 1. Treatment as usual (TAU) 2. TAU plus contingency management (CM; chance to earn prizes or vouchers for submitting negative samples and/or completing goal-related activities)	1.No 2. Yes	RAWC; Months 1, 3, 6, and 9 f/u, 84.2, 81.2, 73.5, and 69.0% f/u rates at months 1, 3, 6, and 9. Tx completion rate not reported.	Quality of life (QOLI) scores over time differed by group, with QOLI scores rising over time in CM participants and remaining stable in TAU. CM achieved greater durations of abstinence, and duration of abstinence was correlated with post treatment QOLI. Abstinence mediated the relationship between treatment condition and QOLI over time.
Rawson et al., 2002 [62]	120 patients in methadone maintenance program with CD (SCID). Mean age 43 years of age, 32% African American, 26% Hispanic.	16-weeks: 1. Contingency Management (CM; vouchers for stimulant-free urine samples; three samples per week and meet briefly with the CM technician) 2. CBT group (CBT; three 90-min group sessions each week, for 16 weeks). 3. CM plus CBT groups (CM-CBT; separate sessions) 4. Treatment as usual (TAU; methadone maintenance clinic)	1. Yes 2. Yes 3. Yes 4. No	RAAT; 17, 26 and 52 weeks, 80% follow-up rate, tx completion not reported.	Two CM groups had superior urine analysis results compared to CBT and TAU at 16 weeks. At week 17 all groups but TAU evidenced reduced rates of cocaine use. At 26 and 52 week f/u CBT showed improvement, gaining equivalence to CM groups in urine analysis and cocaine use.
Rohsenow et al., 2000 [34]; Rohsenow et al., 2001 [63]	128 recruited patients in private substance abuse treatment facilities with CD (SCID). Mean age 28 years, 31% female, 8% African American.	Up to eight 45-min individual sessions held three to five times per week: 1. Meditation-relaxation training (MRT; Control). 2. Cocaine specific coping skills treatment (CST).	1. Yes 2. Yes	RAWC; 3-, 6- and 9-month f/u., 79% f/u rate, 84% tx completion (*N* = 108).	CST participants who relapsed had significantly fewer cocaine use days than did the control group during the first 6 months f/u, no differences over 9-month f/u. CST drank alcohol more frequently during 6 months f/u than MRT. No differences in heavy drinking days. No interaction of treatment was found with gender, education, route of administration, drug use severity, sociopathy, or depression.
Rosenblum et al., 1995 [64]; Rosenblum et al., 1999 [65]	198 methadone patients with CD (SCID), stabilized methadone dose without psychosis or medical condition. Mean age 38 years, 43% female, over 50% Hispanic.	26 weeks: 1. Cognitive behavioral therapy (CBT; Matrix model; 5 days per week, 30 min individual and 45 min. Group sessions. During week 1–4, 3 individual and 2 group sessions, at week 5, 2 individual sessions and 3 group sessions) 2. Low intensity therapy (LIT; weekly group)	1. Yes 2. Yes	RAAT; 48-week f/u. 97.5% 6- month f/u rate, 90.4% 15-month f/u rate. 60% tx completion.	Both groups showed significant and equivalent reductions in cocaine use. Completing tx and lower cocaine severity at baseline were associated with lower proportion of cocaine-positive urines over f/u. High-severity patients improved more in CBT compared to LIT. Positive outcomes for therapy completers relative to non-completers increased over time.
Volpicelli et al., 2000 [66]	87 mothers with CD (ASI) without psychosis, homicidal or suicidal, medical condition, or opioid dependence. Mean age 32 years, 100% female, 97% African American.	Group therapy sessions (GDC) available 5 days per week, expected to attend 2 sessions per week, plus: 1. Case management (CM; 1 15-min session weekly) 2. Psychologically enhanced program (PET access to parenting classes, GED classes, access to a staff psychiatrist, and unlimited access to an individual therapist)	1. No 2. Yes	RAAT; 12-month f/u, 50% completed PET, and 40% completed CM. f/u rates not reported.	Program retention was better for patients in PET. Mean number of days of cocaine use decreased from baseline in both groups, and PET had fewer days of cocaine use at 12-month f/u than CM.
Weinstein, et al. (1997) [67]; Gottheil et al. (1998) [68].	447 referred patients with CD (screened via Risky AIDS Behavior Inventory), and not psychotic, suicidal or cognitively impaired. Mean age of 32 years, 44% female, 93% African American.	Weekly sessions for 12 weeks. 1. Individual counseling (IC, 1 h; supportive, expressive, problem focused) 2. Individual counseling (1 h) plus 1 weekly group session (1 h) (IC-G) 3. Intensive Treatment group (IT; 3 –hours of group, 3 times per week).	1. No 2. No 3. No	RAAT; 6-month and 9-month f/u, 70% of IC completed f/u, 72% of IC-G completed f/u and 70% of IT completed f/u. 20% of IC and IC-G completed tx, and 32% of IT completed tx.	IT evidenced improvement on addiction severity, depression and psychiatric symptoms at end of tx. Regardless of group, at 9-month f/u participants who remained in treatment longer evidenced fewer drug problems, positive drug screens, better employment and fewer psychiatric problems. No significant differences between groups at 6-month or 9-month follow-up.

^a^DSM criteria used unless otherwise noted. ^b^*RAAT* Random Assignment to Active Treatment, *RAWC* Random Assignment with Control, *PPWC* Pre-Post with Comparison Group (matched or otherwise). ^c^*AD* Alcohol Dependence, *CD* Cocaine Dependence. Articles included in the review utilized interview diagnosed screening materials (i.e., SCID) to identify drug abuse or dependence. Articles included in this table utilized a control group

### Group-based methamphetamine use treatments for adults

Only five treatment studies were identified that examined group treatments for methamphetamine use (see Table 3). Three studies found longer periods of abstinence for the group treatment (CM or drug+CM) than for TAU or non-CM conditions. The first study conducted by Rawson and colleagues compared matrix model (MM) with TAU in eight community outpatient settings [71]. The MM consisted of CBT groups, family education groups, social support groups, and individual counseling sessions along with weekly urine screens for 16 weeks. Participants in the MM condition attended more sessions, stayed in treatment longer, had more than twice as many contacts, evidence longer abstinence and greater self-reported psychosocial functioning relative to the TAU group. However, these significant differences did not persist 6 months later at follow-up.

**Table 3:** Summary of evidence-based methamphetamine use group treatment for adults

Study	Patient Characteristics^**a**^	Treatment Description	Manual	Study Characteristics^**b**^	Results
Jaffe et al., 2007 [70]	145 methamphetamine-dependent (DSM-IV) gay and bisexual males. Mean age 37 years, 100% male, 80% White,12% Hispanic.	1. CBT (control condition; 90-min group, 48 session available) 2. Contingency management (CM) (Participants did not need to attend CBT sessions they were only provided vouchers for attending clinic visits) 3. CBT + CM (90-min groups + opportunity to earn vouchers) 4. “Gay Specific” CBT (90-min group session occurred three times per week)	1. Yes 2. No 3. Yes 4. Did not report	RAWC; No follow up reported.	Participants’ in the “Gay Specific” CBT condition reported the most rapid decline in levels of methamphetamine use relative to the other 3 treatment conditions. Participants’ in the control condition reported the highest rates of methamphetamine use.
Rawson et al., 2004 [71] Rawson et al., 2002) [62].	978 treatment seeking individuals with methamphetamine abuse or dependence (DSM-IV checklist), without medical detoxification from opioids/alcohol/ other drugs. Mean age 33 years, 55% female, 18% Hispanic.	1. Treatment as usual (TAU; contact with site 1–13 h. per week). 2. Matrix Model (MM; 16-weeks; 36 cognitive behavioral therapy groups, 12 family education groups, 4 social support groups, 4 individual counseling sessions, combined with weekly testing for cocaine, methamphetamine, opiates, cannabis and benzodiazepines. 12-Step meetings encouraged.	1. No 2. Yes	RAWC; 6-month f/u, 81% f/u rate at post-tx, 86% f/u rate at 6-months. Mean tx contact in TAU was 12 sessions, mean tx contact for Matrix group was 27 sessions.	MM participants attended more sessions, stayed in treatment longer, provided more methamphetamine-free urine samples during the treatment and had longer periods of abstinence than TAU. Drug use and functioning at discharge and 6-month f/u indicate significant improvement by participants in all sites and conditions, but the superiority of MM approach did not persist at f/u.
Roll et al., 2013 [72]	118 participants with methamphetamine dependence (DSM-IV checklist) without a recent suicide attempt (past 30 days), suicidal ideation, parole status or history of violent criminal behavior, and medical condition that could interfere with treatment	1. Standard psychosocial treatment (ST) based on the Matrix Model 2. ST + 1 month of CM 3. ST + 2 months of CM 4. ST + 4 months of CM	1. Yes 2. Yes 3. Yes 4. Yes	RAWC; Retention rates were: 37% completed ST, 67% completed 1 month of CM, 53% completed 2 months of CM, 76% completed 4 months of CM. Post-treatment 4 month f/u; 42% for the ST condition, 43% for the 1 month of CM, 62% for the 2 month of CM, and 64% for the 4 month CM.	The standard treatment group was significantly different from the 4-month CM condition. The group conditions remained abstinent as follows: 3.4% of the ST condition, 13.3% of 1 month of CM condition, 20.0% of the 2-month condition, and 34.5% of the 4 month CM condition. Participants in the 4-month CM condition were more likely to attend f/u session and submit negative urine samples than participants in the ST condition. Results indicated that attendance, consecutive days of methamphetamine abstinence, and the number of participants who remained 100% or 80% abstinent throughout the trial increased as the duration of CM went up.
Shoptaw et al., 2006 [73]	229 treatment seeking individuals with methamphetamine abuse or dependence (SCID) and without medical condition, current treatment with a selective serotonin reuptake inhibitor, a psychiatric condition, or dependence on opioids, cocaine, alcohol, or benzodiazepines. Mean age 33 years, 38% female, 23% Latino.	2-week non-medication baseline with 12 weeks of medication tx and: 1. Sertraline plus Contingency Management (S-CM; 4 weekly relapse prevention groups, three times per week) 2. Sertraline-only (S) 3. Placebo medication plus CM (P-CM) 4. Placebo medication (P)	1.Yes 2. No 3. Yes 4. No	RAWC; post-treatment f/u; 50.7% completed all 14 weeks of the trial.	No effects for sertraline or CM in reducing methamphetamine use were observed. S had significantly poorer retention and produced significantly more adverse events than P-CM or P. More participants in CM conditions achieved three consecutive weeks of methamphetamine abstinence than those in non-CM conditions.

^a^DSM criteria used unless otherwise noted. ^b^RAAT Random Assignment to Active Treatment, RAWC Random Assignment with Control, PPWC Pre-Post with Comparison Group (matched or otherwise). Articles included in the review utilized interview diagnosed screening materials (i.e., SCID) to identify drug abuse or dependence. Articles included in this table utilized a control group

Shoptaw et al. (2006) [73] compared four groups for treating methamphetamine dependence sertraline + CM, sertraline only, placebo + CM, and placebo [73]. Additionally, all participants attended a relapse prevention group conducted three times a week over a 14-week period. Findings provided support for the efficacy of CM for amphetamine use disorders. Group treatment (CM or drug + CM) was more effective for sustaining longer periods of abstinence relative to TAU or non-CM conditions. Roll et al. [72] found that effects of CM relative to TAU became larger as the duration of CM increased. Jaffe et al. [70] evaluated a culturally tailored intervention for 145 methamphetamine dependent gay and bisexual males. Participants in the Gay Specific CBT condition reported the most rapid decline in levels of methamphetamine use relative to standard CBT, CBT + CM, suggesting benefits for culturally appropriate group methamphetamine interventions.

### Group-based marijuana use treatments for adults

Two studies examining group treatments for adults with marijuana use disorders were identified (see Table 4). Both studies were conducted by the same research group, utilizing the same inclusion criteria for marijuana use (50 times in 90 days). The studies examined group relapse prevention (RP) [76], specifically designed for adult marijuana users. The first trial [75] (*n* = 212) comparing relapse prevention to a social support group found participants in both group treatment conditions did well overall, with two-thirds (65%) reporting abstinence of marijuana use for 2 weeks after session 4 or the quit date and 63% reporting abstinence during the last 2 weeks of treatment. Gender differences emerged; no differences between group treatments were found for women, but men in the relapse prevention group reported reduced marijuana use at the 3-month follow-up compared to men in the social support group.

**Table 4:** Summary of evidence-based marijuana use group treatment for adults

Study	Patient Characteristics^**a**^	Treatment Description	Manual	Study Characteristics^**b**^	Results
Stephens et al. 2000 [74]	291 recruited individuals using marijuana more than 50 times in past 90 days (questionnaire screening), without severe psychological distress, psychosis, suicidal, cognitive impairments or formal treatment for marijuana use. Mean age 34 years, 23% female, 95% Caucasian.	1. Relapse prevention support group (RSPG; 14-sessions, 2 h each, over 18 weeks). 2. 2-session motivational interviewing (MI; Drinkers Check Up; 2 90-min sessions). 3. 4-month delayed treatment control (DTC)	1. Yes 2. Yes 2. No	RAWC; 1-, 4-, 7-, 13-, and 16-month f/u. 88% RSPG f/u rate, 92% MI f/u rate. Average number of RPSG treatment sessions was 8.42 out of 14. 86% MI tx completion.	Marijuana use, dependence symptoms, and negative consequences were reduced significantly in relation to pretreatment levels at 1-, 4-, 7-, 13-, and 16-month f/u. RPSG and MI evidenced greater improvement than DTC at the 4-month f/u. No significant differences between RPSG and MI outcomes at any f/u.
Stephens et al., 1994 [75]	212 recruited participants reporting using marijuana more than 50 times in 90 days (questionnaire screening), without other substance abuse or dependence, psychosis, or current treatment for marijuana use. Mean age 32 years, 24% female, 95% Caucasian.	10 2-h sessions: 1. Relapse prevention support group (RPSG) 2. Social Support Group (SSP)	1. Yes 2. No	RAAT; 1–3- 6- and 9-and 12-month f/u. 69% treatment completion, 78% f/u rate.	Men in RP were more likely than men in SSP to report reduced marijuana use without problems at 3-month f/u. No other differences between groups emerged.

^a^DSM criteria used unless otherwise noted. ^b^*RAAT* Random Assignment to Active Treatment, *RAWC* Random Assignment with Control, *PPWC* Pre-Post with Comparison Group (matched or otherwise). Articles included in the review utilized interview diagnosed screening materials (i.e., SCID) to identify drug abuse or dependence. Articles included in this table utilized a control group

A second trial [74] randomized participants to 14 sessions of group RP enhanced with cognitive behavioral skills training, two sessions of motivational interviewing (MI) with feedback and advice on cognitive behavioral skills (modeled after the Drinkers Check-up) [77], or a 4-month delayed treatment control (DTC) group which consisted of the RP group or individual MI treatment of the participants choosing. Compared to individuals randomly assigned to the DTC condition, participants in the group RP and individual MI conditions evidenced a significantly greater reduction in marijuana use and related problems over 16-month follow-up. However, examination of participants’ reactions to DTC assignment indicated that participants who felt that changing their marijuana use was their own responsibility were more likely than those who did not to change their use patterns without treatment engagement.

### Group-based opiate use treatments for adults

Five group treatment studies for opioid use were identified (see Table 5). Two studies compared the effectiveness of pharmacotherapy plus group therapies [79–81] to pharmacotherapy alone in samples of opioid dependent persons, and both found that adding group treatment improved outcomes. The first study compared Naltrexone with monthly medical monitoring visits to an enhanced group condition (EN) consisting of Naltrexone plus a Matrix Method (MM) [79]. MM consisted of hourly individual sessions, 90-min CBT group, and 60 min of cue-exposure weekly for weeks 1–12; hourly individual sessions and CBT group sessions for weeks 13–26; and 90-min social support group sessions for weeks 27–52. Results found that EN participants took more study medication, were retained in treatment longer, used less opioids while in treatment, and showed greater improvement on psychological and affective dimensions than Naltrexone only participants. No difference by treatment condition was found at 6- and 12-month follow-ups. Similarly, Scherbaum et al. [80] compared routine Methadone Maintenance Therapy (MMT) with routine MMT plus group CBT psychotherapy (20 90-min sessions for 20 weeks). MMT plus group CBT participants showed less drug use than participants in the MMT group (i.e., control group). In contrast, a higher dose of group therapy provided without methadone maintenance was less effective for heroin use than was a lower dose of group therapy with methadone maintenance (Sees et al. [81]. This suggests that the combination of pharmacotherapy and group therapy for opioid use is optimal.

**Table 5:** Summary of evidence-based opiate use group treatment for adults

Study	Patient Characteristics^**a**^	Treatment Description	Manual	Study Characteristics^**b**^	Results
Des Jarlais et al., 1992 [78]	104 individuals who were using heroin intranasally, without using more than 60 injections in past 2 years (questionnaire screening). Mean age 27 years, 31% female, 27% African American, 24% Hispanic.	All participants received information about AIDS, and HIV antibody test counseling: 1. Social learning AIDS/drug injection treatment program (4 sessions, 60–90 min, over 2 weeks) 2. Control condition	1. Yes 2. No	RAWC; 8-month f/u. 80% f/u rate, tx completion rate not reported.	Control participants reported higher rates of drug injection over f/u.
Rawson et al., 2001 [79]	81 recruited detoxified individuals meeting DSM-IV criteria for opioid dependence (diagnostic screening measure not reported). Mean age 33 years, 45% female, 80% Caucasian.	1. Standard treatment (ST; Naltrexone, with monthly medical monitoring visits). 2. Enhanced group (EN; Matrix Method; Naltrexone plus: Week 1–12 consists of 60 min individual sessions 1x week, 90 min. CBT group, and 60 min cue-exposure; Week 13–26 consist of individual session semi-weekly, and CBT group sessions, and Week 27–52 consist of 90 min social support group sessions).	1. No 2. Yes	RAWC; 6-, 12-month f/u. 84% f/u and 87% f/u at 6-month for ST and EN respectively. 74 and 79.5% f/u at 12-month for SN and EN respectively. Tx completion not reported	EN group participants took more study medication, were retained in treatment longer, used less opioids while in treatment and showed greater improvement on a number of psychological/affective dimensions. No significant group differences at 6- or 12-month f/u
Scherbaum et al., 2005 [80]	73 patients at methadone maintenance treatment with opiate addiction (SCID) and no severe psychiatric condition, psychosis, and organic brain syndrome, serious medical, legal, or social problems. Mean age 30 years, 27% female, 96% reported at least 1 parent of German origin.	6-months: 1. Local routine MMT 2. Routine MMT plus group CBT psychotherapy (MMT-CBT; 20 90-min sessions, 20 weeks)	1. No 2. Yes	RAWC; 6-month f/u. f/u rate not reported. 63% of MMT-CBT and 59% of MMT completed tx.	MMT-CBT showed less drug use than MMT, statistically significant at post treatment and 6-month f/u.
Sees et al., 2000 [81]	179 recruited individuals with opioid dependence (DIS). Mean age 39 years, 47% female, 23% African American in DT, 31% African American in MMT, 15% Hispanic in DT, 8% Hispanic in MMT.	1. Methadone maintenance therapy (MMT; 2 h psychosocial therapy during 1st 6 months, up to 14 months followed by 2-month detoxification) 2. 180-day Methadone assisted detoxification (DT; 2 h of psychosocial group therapy per week, 14 weekly substance abuse education sessions, 1 h. of cocaine group therapy for 6 months, weekly individual therapy and 8 months of non-methadone aftercare sessions after 1st 6 months)	1. Yes 2. Yes	RAAT; 12-week f/u, 74% f/u rate, 86% tx completion rate.	MMT resulted in greater treatment retention and less heroin use compared to DT. Cocaine was related to study dropout in MMT. MMT resulted in lower rate of drug related HIV risk behaviors and lower severity score for legal status. No differences between groups in employment, family functioning, alcohol use.
Shaffer et al. (1997) [22]	61 referred patients to a methadone maintenance clinic (screened via “standard assessment battery”), without physical or medical inability to participate in yoga. Mean age 36 years, 41% female, 82% Caucasian.	22 75-min sessions. All pts. received methadone treatment and individual therapy. 1. Psychodynamic group therapy 2. Hatha yoga group	1. Yes 2. Yes	RAAT; 6-month f/u. f/u rate not reported. 69% tx completion.	Longer participation in treatment was associated with reduction in drug use and criminal activity. No difference on any measures between two treatments.

^a^DSM criteria used unless otherwise noted. ^b^*RAAT* Random Assignment to Active Treatment, *RAWC* Random Assignment with Control, *PPWC* Pre-Post with Comparison Group (matched or otherwise). Articles included in the review utilized interview diagnosed screening materials (i.e., SCID) to identify drug abuse or dependence. Articles included in this table utilized a control group

Shaffer et al. [22] compared psychodynamic group therapy with a hatha yoga group. All participants received methadone maintenance and individual therapy. No differences between two treatment conditions were found. For all participants, longer participation in treatment was associated with reduction in drug use and criminal activity. Lastly, Des Jarlais et al. [78] compared a group social learning AIDS/drug injection treatment program (4 sessions, 60–90 min, over 2 weeks) to a control condition. All participants received information about AIDS and HIV antibody test counseling. Compared to control participants, intervention participants reported lower rates of drug injection over time.

### Group treatments for mixed SUD for adults

Nine treatment studies were identified that targeted mixed substance use with group treatments (see Table 6). Three involved CBT. Downey et al. [82] compared group CBT plus individual CBT to group CBT plus vouchers in a sample of 14 polysubstance users (cocaine and heroin) maintained on buprenorphine. The study was significantly underpowered and they found no significant differences on treatment outcomes. Marques and Formiogioni [84] compared individual CBT to group CBT in a sample of 155 participants with alcohol and/or drug dependence. They found that both formats resulted in similar outcomes, with higher compliance in the group CBT participants (66.7% compliance with treatment). Rawson et al. [87] compared three 16-week treatments: CM, group CBT, and CM plus group CBT, among 171 participants with cocaine disorder or methamphetamine abuse. They found that CM produced better retention and lower rates of stimulant use than CBT during treatment, but CBT produced comparable longer-term outcomes.

**Table 6:** Summary of evidence-based mixed sud group treatment for adults

Study	Patient Characteristics^**a**^	Treatment Description	Manual	Study Characteristics^**b,c**^	Results
Downey et al., 2000 [82]	14 buprenophrine maintained poly-drug users (cocaine plus heroin). (SCID). Mean age 40 years, 39% female, 35% Caucasian.	18 weeks: 1. Individual CBT (6 sessions) plus 12 session (weekly) group therapy (CBT; relapse prevention) 2. CBT based plus vouchers (VBRT)	1. Yes 2. Yes	RAAT; post-test at end of 18-week tx; 37% tx completion/ f/u rate in CBT; 65% tx completion/f/u VBRT.	No significant differences on treatment outcome. Among the subsample that produced one or more poly-drug free urine results, VBRT participants had significantly increased cocaine abstinence.
Greenfield et al., 2007 [52]	13 (for pilot) and 31 (in trial) recruited patients with SUD (other than nicotine; SCID), substance use within 60 days of baseline, and no need for medical detoxification, mandate to treatment, psychosis, PTSD, concurrent self-help group treatment. Mean age 58 years for GDC and 45 for WRG, 100% female, predominantly Caucasian.	12 weeks, 90-min sessions, 1x per week: 1. Group Drug Counseling (GDC; mixed gender; 12 weeks) 2. Women’s Recovery Group (WRG; author)	1. Yes 2. Yes	RAWC; 6-month f/u. 87% f/u rate, 78% tx completion.	Pilot testing of WRG evidenced significantly greater reductions in average drinks/drinking day than GDC at 6-month f/u. WRG was equally effective as mixed-gender GDC in reducing substance use during the 12-week in-treatment phase, but demonstrated significantly greater improvement in reductions in drug and alcohol use over the f/u compared with GDC. Women were significantly more satisfied with WRG than GDC
Margolin et al., 2003 [83]	90 HIV-seropositive, methadone-maintained injection drug users with opioid dependence, and abuse or dependence on cocaine (screened at intake, utilizing Addiction Severity Index). Mean age 41 years, 30% female, 48.9% African American, 15.6% Hispanic.	6-months of methadone maintenance plus: 1. HIV Harm Reduction Program (HHRP; twice weekly, 2-h groups) 2. Active control that included harm reduction components recommended by the National AIDS Demonstration Research Project (six sessions).	1. Yes 2. Yes	RAWC; 6- and 9-month f/u. 71% 6-month f/u rate, 70% 9-month f/u rate. 64.4% tx completion.	Both groups showed reductions in risk behaviors. HHRP evidenced less use of illicit opiates and more adherence to antiretroviral medications; at follow-up, they had lower addiction severity scores and were less likely to have engaged in high risk behavior compared to control.
Marques & Formigioni (2001) [84]	155 recruited alcohol and/or drug dependent patients (standardized assessment interview). Mean age of drug dependent patients 25 years Mean age of AD patients 41 years, 8% female. No ethnicity data reported.	17 sessions over 8 months (1 session per week during Month 1–2, 1 session every 2 weeks in Month 3–5, 1 session per month during Month 6–8. 1. Individual CBT (IT; structured) 2. Group CBT (GT; structured)	1. Yes 2. Yes	RAAT; 15-month f/u. 66% f/u rate in IT, 70% f/u rate in GT. IT attended average of 7 sessions, GT attended average of 8 sessions.	At follow- up the two formats presented similar outcomes, higher compliance in GT (66.7%)
McKay et al., 2005 [85]	359 referred patients with AD or CD (SCID). Mean age 42 years, 17% female, 77% African American.	12-week continuing care interventions: 1. weekly telephone monitoring and counseling combined with a support group in the first 4 weeks (TEL); 2. twice-weekly individualized relapse prevention (RP) 3. twice-weekly standard group counseling (STND; 12 step).	1. Yes 2. Yes 3. Yes	RAAT; 3, 6, 9- and 12-months f/u. 90% f/u rates. The average number of sessions was 14.12 in STND, 14.41 in RP and 10.94 in TEL.	Days of abstinence were higher in STND than TEL. Higher scores on a composite risk indicator indicated higher abstinence rates in STND than TEL and lower composite risk scores indicated higher abstinence rates in TEL than STND.
Nemes et al., 1999 [86]	412 patients in a therapeutic community with multiple drug/alcohol use dependencies/abuse (SCID. Mean age “mid-thirties”, 23% SC females, 33% AP females, primarily African American.	12-month program (inpatient and outpatient): 1. Standard Care (SC, 10 months inpatient, 2 months outpatient) 2. Abbreviated program (AP, 6 months inpatient, 6 months outpatient)	1. No 2. No	RAWC; 6-month f/u. 93% f/u rate. SC completed average of 8.2 months of program; AP completed average of 8.6 months of program.	Both groups had reductions in arrests and drug use. No significant differences between groups.
Rawson et al., 2006 [87]	171 recruited individuals with CD or methamphetamine abuse (SCID), and no AD or benzodiazepine dependence, or court mandated to treatment. Mean age 36 years, 24% female, 32% African American.	16-weeks: 1. Contingency Management (CM; vouchers for stimulant-free urine samples; three urine samples per week and meet briefly with the CM technician) 2. CBT group (CBT; three 90-min group sessions each week, for 16 weeks). 3. CM plus CBT groups (CM-CBT; separate sessions)	1. Yes 2. Yes 3. Yes	RAAT; Baseline and weeks 17, 26 and 52 f/u. 81% f/u rate. 60% CM completed tx, 59% CM-CBT completed tx, and 40% CBT completed tx.	CM produced better retention and lower rates of stimulant use during the study. Stimulant use was reduced from baseline levels at all f/u points for all groups and urinalysis data did not differ between groups at f/u. CM produced evidence of efficacy during treatment application, but CBT produced comparable longer-term outcomes. There was no evidence of an additive effect in CM-CBT.
Schottenfeld et al., 2000 [88]	117 patients with opioid dependence and CD or cocaine abuse (SCID) without psychosis and not suicidal or pregnant. Mean age 34 years, 49% female, 64% Caucasian.	In addition to maintenance medications- 24 weeks of: 1. Group Drug Counseling (GDC; weekly, 1-h group DC sessions). 2. Community Reinforcement Approach (CRA; met in individual sessions with a CRA therapist twice weekly during the first 12 weeks and then weekly during the following 12 weeks).	1. Yes 2. Yes	RAAT; 9-week ff/u. No f/u rate reported. Tx completion for GDC was 59.6 and 61.7% for the CRA.	There were no significant differences in retention or drug use. The total number of hours and average hours per week engaged in nondrug-related activities was higher for CRA patients who achieved abstinence from opioids, cocaine, or both combined.
Smith et al., 1999 [89]	383 inpatient veterans, meeting AD, CD or amphetamine dependence (Semi-structured interview). Mean age 40–50 years, 0% female, 11–46% of participants in each group were African American.	Between 21 and 28 days of treatment: 1. Standard treatment program (STP; daily group counseling, family outreach, 12-step program introduction, four 2-h. sessions for family) 2. Enhanced treatment program (ETP; 10 h. per week, twice weekly groups on relapse prevention and interpersonal counseling)	1. No 2. Yes	1st cohort completed STP; 2nd cohort completed ETP; 3- and 12-month f/u. 92% f/u rate at 3-month and 83% f/u at 12-month. 80% tx completion.	ETP evidenced enhanced abstinence rates at 3-month and 12-month follow-up compared to STP, regardless of type of drug use.

^a^DSM criteria used unless otherwise noted. ^b^*RAAT* Random Assignment to Active Treatment, *RAWC* Random Assignment with Control, *PPWC* Pre-Post with Comparison Group (matched or otherwise). ^c^*AD* Alcohol Dependence, *CD* Cocaine Dependence, *SUD* Substance Use Disorder. Articles included in the review utilized interview diagnosed screening materials (i.e., SCID) to identify drug abuse or dependence. Articles included in this table utilized a control group

Two studies involved Group Drug Counseling (GDC). Greenfield et al. [52] compared a group drug counseling (GDC) (mixed gender) to a women’s recovery group (WRG) that both met weekly, for 12 weeks, for 90-min sessions among 44 participants that had a substance use disorder other than nicotine. WRG evidenced significantly greater reductions in drug and alcohol use over the follow up compared with GDC. Schottenfeld et al. [88] compared GDC (weekly, 1-h group sessions) to a community reinforcement approach (CRA; twice weekly sessions for the first 12 weeks and then weekly the following 12 weeks) among 117 patients with an opioid and cocaine use disorder. There were no differences in retention or drug use.

Remaining studies examined other interventions. Margolin et al. [83] compared an HIV Harm reduction program (HHRP) that met twice weekly for 2 h to an active control group that met six times in a sample of 90 HIV-seropositive methadone-maintained injection drug users with opioid dependence, and abuse or dependence on cocaine. At follow up, they had lower addiction severity scores and were less likely to have engaged in high risk behaviors compared to control. McKay et al. [85] compared weekly phone monitoring and counseling plus a support group in the first 4 weeks (TEL), twice-weekly individualized relapse prevention, and twice-weekly standard group counseling (STND) among 259 referred participants with alcohol use disorder or cocaine disorder. STND resulted in more days abstinent than TEL. Nemes et al. [86] compared a 12-month group program (10 months inpatient and 2 months outpatient) to an abbreviated group program (6 months inpatient, 6 months outpatient) among 412 patients with multiple drug/alcohol use disorders. Results indicated that both groups had reduction in arrests and drug use. There were no significant difference between groups. Lastly, Smith et al. [89] compared a standard treatment program (STP, daily group counseling, family outreach, 12-step program introduction, four 2 h sessions for family) to an enhanced treatment program (ETP; twice weekly group on relapse prevention and interpersonal violence in additional to all STP components) among 383 inpatient veterans meeting for an alcohol, cocaine, or amphetamine use disorder. Results indicated that ETP had enhanced abstinence rates at 3-month and 12-month follow up compared to STP, regardless of type of drug use.

### Group Treatments for SUD and Co-Occurring Psychiatric Problems

Individuals with psychiatric distress are at high risk for comorbid SUD [90]. Ten randomized controlled studies meeting our inclusion criteria examined the efficacy of group therapy for SUD and co-occurring psychiatric problems (see Table 7). Three studies described group treatment of SUD and co-occurring DSM-IV Axis II disorders [18, 91, 96], three studies examined group treatment of drug abuse and co-occurring DSM-IV classified Axis I disorders [92, 93, 99], one study explored group drug abuse treatment and co-occurring psychiatric problems among homeless individuals without limiting to DSM-IV Axis I or Axis II diagnoses [97], and one study focused on group drug treatment among individuals testing positive for HIV [98]. Within this diverse set of RCTs, participants generally included individuals diagnosed with any form of SUD; however, some studies focused specifically on individuals using cocaine [91, 97] or cocaine/opioids [98].

**Table 7:** Summary of evidence-based drug abuse disorders and co-occurring psychiatric problems group treatment for adults

Study	Patient Characteristics^**a**^	Treatment Description	Manual	Study Characteristics^**b,c**^	Results
Compton et al., 2000 [91]	996 recruited outpatient cocaine users with and without antisocial personality disorder (ASPD) and major depression (DIS). Mean age 39 years, 39% female, 92% African American.	Two 15-min sessions, plus 4 peer-administered 2-h sessions: 1. Standard Intervention (SI; developed by NIDA Cooperative Agreement Final Cohort sites; 2 15-min sessions) 2. Enhanced Intervention (EI; SI plus 4-peer administered 2-h sessions)	1. Yes 2. Yes	RAAT; 3-month follow-up. 88%, f/u rate, 100% participation in SI, 69% tx completion in EI.	All groups improved significantly in: crack cocaine use, injection drug use (IDU), number of IDU sex partners and overall number of sex partners. Stratified by psychiatric status, ASPD was associated with significantly less improvement in crack cocaine use. When examining the standard and peer groups separately, no consistent differences in the association of psychiatric comorbidity with outcome were evidenced.
DiNitto et al., 2002 [92]	97 recruited inpatients at chemical dependency treatment program, with Axis I disorder (ASI, Addiction Severity Index). Mean age 33 years, 53% female, 28% African American.	28-days of treatment: 1. Treatment as usual (TAU; Inpatient chemical dependency services) 2. Good Chemistry Group (GCG; TAU plus psychoeducational group therapy; 9 60-min sessions; 3 times a week; repeated for 15 months)	1. No 2. Yes	RAWC; 1-. 2- and 3-month f/u. 86% f/u rate. Average treatment 25.6 days for GCG and 26.3 days for TAU.	No significant differences between groups.
Fisher & Bentley (1996) [18]	38 referred inpatient and outpatient with SUD and personality disorder (SCID). Mean age 37 years, 24% female, 61% African American.	45-min sessions, 3x per week, for 4 weeks: 1. Disease-recovery group (DRG; acceptance of substance abuse as a chronic and progressive disease) 2. CBT-group 3. Group treatment as usual (Control)	1. Yes 2. Yes 3. No	RAWC; Full sample completed pre and post-test assessments (e.g., 100% tx completion and f/u rate).	DRG and CBT evidenced improved social/family relations compared to control. CBT more effective than DRG group in reducing alcohol and improving social/family function and enhancing psychological function.
Jerrell et al., 1995 [93]; Jerrell et al., 1997 [94]	132 recruited outpatients with psychotic or Axis I disorder and SUD (DIS) and poor work history; eligibility for public assistance, poor basic living skills, poor social support, or poor social skills. Excluded based on cognitive impairment, personality disorder and medical disabilities. Ages 28–59, 23% female, no ethnicity data provided.	1. Twelve-step group (TS; one to several meetings per week; structured) 2. Behavioral Skills group (BS; Social and Independent Skills program; one group per week) 3. Intensive case management (Program for Assertive Community Treatment; as needed 5 day/week)	1. Yes 2. Yes 3. Yes	RAAT. 18-month f/u. No data provided on f/u rate or tx completion rate.	BS and ICM evidenced significant decreases in schizophrenia, depression and mania symptoms compared to TS. BS also evidenced significant decreases in drug and alcohol use compared to TS. Compared to men, women had higher functioning scores, more psychiatric symptomatology, and greater reductions in use of acute treatment services used over the 6-month f/u.
Lehman et al., 1993 [95]	54 patients with SUD and schizophrenia or affective disorder (SCID). Mean age 30 years, 26% female, 79% African American.	5 1-h sessions and 2 months of intensive case management: 1. Treatment as usual (Control; Community mental health center and rehabilitation services) 2. Being sober group, plus group and intensive case-management (ICM-G)	1. No 2. Yes	RAWC; 1 year f/u, No f/u rate reported. 20% average tx attendance.	One-year follow-ups detected no significant differences between ICM-G and Control (treatment as usual).
Linehan et al. 1999 [96]	27 referred from community care, with borderline personality disorder and SUD (opiates, cocaine, amphetamines, sedatives, hypnotics, anxiolytics, or polysubstance use; SCID and International Personality Disorders Exam). Mean age 30 years, 100% female, 78% Caucasian.	Weekly 1-h individual sessions; 2-h group sessions; coaching as needed for 12 months: 1. Treatment as usual (TAU; outpatient psychotherapy or community care). 2. Dialectical Behavior Therapy Group modified for substance use (DBT).	1. No 2. Yes	RAWC; 16-month f/u; 66% f/u rate, 70% tx completion rate.	DBT evidenced greater reductions in drug use compared to TAU throughout treatment and at f/u. DBT evidenced significantly higher tx retention compared to TAU, and greater global adjustment at follow-up compared to TAU.
Milby et al., 2004 [97]	141recruited cocaine-dependent homeless individuals and co-occurring non-psychotic mental disorder (DSM-III-R checklist). Mean age 38, 72% male, 83% African American.	All participants received: Phase I (8 weeks day treatment, 5 days per week, 5.5 h per day; highly structured) and Phase II (16 weeks of weekly group therapy, individual counseling 1 time per week). 1. Day treatment only (DT) 2. Day treatment plus abstinent-contingent housing and work (DT+)	1. Yes 2. Yes	RAAT; 2-, 6-, and 12-month f/u. At 2-months, 76.3% f/u, at 6-months, 74.5% f/u. 37% tx completion in DT, 77% of DT+ tx completion.	Compared with DT, more DT+ participants established abstinence, maintained abstinence for longer durations, were marginally significantly more likely to lapse, and significantly less likely to relapse. Of all participants who established abstinence and then relapsed, DT+participants relapsed later and were more likely to reestablish abstinence.
Petry et al. (2010) [98]	170 HIV+ patients with cocaine or opioid abuse or dependence over past year (via SCID). Mean age 43 years, 39% female, 44% African American, 32% Hispanic.	Weekly groups for 24 weeks: 1.Contingency management (CM) 2.Twelve step groups (TS)	1. Yes 2. Yes	RAAT; 1-, 3-, 6-, 9- and 12-month f/u; mean attendance 10.8 for CM and 9 for TS.	Compared to TS, CM participants submitted more consecutive drug-free urine specimens; whereas negative urine samples did not vary between groups during treatment or follow-up; CM participants reported fewer HIV-risk behaviors compared to TS during treatment.
Zlotnick et al. (2009) [99]	49 incarcerated women with SUD and full/subthreshold posttraumatic stress disorder (SCID and Clinician Assisted Posttraumatic Stress Disorder Scale-I) without psychotic or organic brain impairment. Mean age 35 years, 100% female, 32.7% African American, 14.2% Hispanic.	6–8-week intervention: 1. Treatment as usual (TAU; 180–240 h of individual and group treatment) 2. Seeking Safety Group (90-min sessions, 3x per week)	1. No 2. Yes	RAWC; 12-week, 3-, and 6-month f/u. 97% 12-week f/u rate, 85% 6-month f/u rate for SS, and 95% 6-month f/u rate for TAU. Women attended average of 15.6 of 25 SS sessions.	Consistent main effects for time but not group by time interaction on key variables (e.g., PTSD, substance use, legal problems). 6 months after release from prison, 53% of the women in both groups reported a remission of PTSD. Some advantages for Seeking Safety were found over TAU during the f/u period (e.g., improvement in psychopathology and recidivism rates).

^a^DSM criteria used unless otherwise noted. ^b^RAAT Random Assignment to Active Treatment, RAWC Random Assignment with Control, PPWC Pre-Post with Comparison Group (matched or otherwise). ^c^AD Alcohol Dependence, CD Cocaine Dependence, SUD Substance Use Disorder. Articles included in the review utilized interview diagnosed screening materials (i.e., SCID) to identify drug abuse or dependence. Articles included in this table utilized a control group

A range of group treatment approaches are represented, including group psychoeducational therapy, group CBT approaches, group DBT, Seeking Safety and CM. DiNitto and colleagues [92] evaluated the efficacy of adding a group-based psychoeducational program entitled “Good Chemistry Groups” to standard inpatient SUD treatment services among 97 individuals with a dual diagnosis of SUD and a DSM-IV Axis I psychological disorder. The nine 60-min Good Chemistry Group sessions were offered 3 times per week for 3 weeks. When compared to standard inpatient treatment, the addition of the psychoeducational group was not associated with any changes in medical, legal, alcohol, drug, psychiatric or family/social problems among participants.

The efficacy of adding a psychoeducational group treatment to standard individual therapy to address HIV risk among cocaine users has also been examined [91]. Participants were randomly assigned to complete the following: 1) individually-administered Standard Intervention developed by the NIDA Cooperative Agreement Final Cohort sites [100] including HIV testing, and pre- and post-HIV testing counseling on risks relating to cocaine use, transmission of STDs/HIV, condom use, cleaning injection equipment, and the benefits of treatment; or) Standard Intervention plus four 2-h peer-delivered psychoeducational groups addressing stress management, drug awareness, risk reduction strategies, HIV education and AIDS. Among the sample of 966 individuals completing the 3-month follow-up, the group psychoeducational treatment was not differentially effective in reducing drug use and HIV risk behavior in comparison to standard treatment alone at 3-months post-baseline, regardless of treatment type, individuals with antisocial personality disorder (ASPD) demonstrated less improvement in crack cocaine use compared to individuals without ASPD or depression.

The following types of group CBT have sustained research evaluation meeting our inclusion criteria to address co-occurring SUD and Axis I or Axis II disorders: 1) group behavioral skills training; 2) group cognitive behavioral therapy; 3) group-based Seeking Safety [51], and 4) group dialectical behavioral therapy. Specifically, Jerrell and Ridgely [93] examined the efficacy of group behavioral skills (BS) training, group-based 12-step facilitation (TS) treatment, and intensive case management among 132 individuals with a dual diagnosis of SUD and another Axis I psychiatric problem over the course of 24-months. Based on the Social and Independent Living Skills program [101], the BS group included one group per week addressing self-management skills designed to enhance abstinence, including medication management, relapse prevention, social skills, leisure activities and symptom monitoring. Relative to participants in TS groups, participants in the BS groups evidenced increased psychosocial functioning and decreased psychiatric symptoms (i.e., schizophrenia, depressive symptoms, mania, drug use and alcohol use) across the 6-, 12- and 18-month follow-up assessments after treatment entry.

Lehman and colleagues’ [95] examination of the efficacy of group CBT for substance abuse compared to TAU among 54 individuals with SUD and either schizophrenia or a major affective disorder revealed no differences between treatment groups over the course of a 1-year follow-up period. More promising findings were reported in Fisher and Bentley’s [18] evaluation of a group CBT and group therapy based in the disease and recovery model (DRM) among 38 individuals with dual diagnosis of SUD and a personality disorder. Groups met three times per week for 12 weeks and were compared to TAU. Individuals in group CBT and group DRM indicated improved social and family functioning compared to TAU, and among those who completed the group in an outpatient setting, CBT was more effective in reducing alcohol use, enhancing psychological functioning and improving social and family functioning compared to DRM and TAU.

Group behavioral therapy plus abstinence contingent housing and work administered in the context of a day treatment program was compared to behavioral group treatment alone among individuals with cocaine abuse/dependence, non-psychotic psychiatric conditions, and homelessness [97, 102]. The group behavioral therapy included 8 weeks of daily treatment (4 h and 50 min per day) of groups addressing relapse prevention training, assertiveness training, AIDS education, 12-step facilitation, relaxation, recreation development, goal setting, and goal planning. Participants also engaged in a process-oriented group as well as individual counseling and urine monitoring and engaged in a weekly 90 min psychoeducational group therapy during months 3–6 following treatment enrollment. Individuals who received contingency-based work and housing were provided with rent-free housing and employment in construction or food service industries after 2 consecutive weeks of abstinence [103]. Relative to BS groups alone, group behavioral day treatment plus contingency management was associated with greater abstinence at 2- and 6-month follow-ups [102] and were less likely to relapse [97], although gains were not maintained at 12-months [104]. Both groups evidenced positive changes in drug use overtime compared to baseline [104].

Zlotnick, Johnston and Najavits [99] evaluated the efficacy of Seeking Safety (SS), in comparison to treatment as usual (TAU) among 49 incarcerated women with substance use disorder (SUD) and full or subthreshold posttraumatic stress disorder (PTSD). SS aims to decrease PTSD and SUD through psychoeducational and present-focused and empowerment-based instruction on coping skills that emphasize abstinence and safety [51]. The SS group treatment included 90-min group sessions held three times per week, that were completed in addition to the 180 to 240 h of group and individual therapy provided in TAU. All participants showed similar improvement on assessments of PSTD, SUD, legal problems and other psychiatric concerns at 12-week, 3- and 6-month follow-ups following prison release. Nonetheless, there was a trend for improved PTSD and continued improvements in psychiatric symptoms at follow-up among participants completing SS compared to TAU. Greater completion of SS sessions was associated with increased improvement in PTSD as well as drug use among women [99].

Dialectical behavioral group therapy (DBT), a CBT-focused treatment for individuals with borderline personality disorder (BPD), has also been evaluated in comparison to TAU among individuals with BPD and co-occurring SUD [96]. Core elements of DBT are manualized [105], and have been evaluated in prior research [106–108]. Techniques center on providing the participant with acceptance and validation while maintaining a continual focus on behavior change, and include the following: mindfulness skills training, behavioral analysis of dysfunctional behavior, cognitive restructuring, coping skills training, exposure-based strategies addressing maladaptive emotions, and behavioral management skills training. DBT was administered through 2 ¼ hour weekly group sessions administered in combination with 60 min of weekly individual therapy and the opportunity for skills-coaching phone calls. Relative to TAU, participants randomly assigned to DBT demonstrated greater reductions in drug use during the 12-month treatment and at the 16-month follow-up assessment, as well as greater gains in adjustment at the 16-month follow-up assessment.

Although contingency management is commonly administered individually, Petry and colleagues [98] examined the efficacy of weekly 60-min group-based contingency management (CM) for reinforcing health behaviors and HIV-positive individuals with cocaine or opioid disorders (*N* = 170) in comparison to 12-step facilitation (TS) over the course of a 24-week period. Overall, participants in CM were more likely than those in TS to submit consecutive drug-free urine specimens, although the overall proportion of drug-free specimens did not vary between groups during treatment or over the follow-up period. Notably, during treatment, group CM was associated with greater reductions in HIV-risk behaviors as well as overall viral load compared to TS; although effects were not maintained over the follow-up period.

Across these studies, many trials showed positive gains for both group treatments examined [18, 97, 98], or no difference between groups when examining the benefit of adding group treatment to existing TAU [91, 92, 95, 99]. However, one study demonstrated greater reductions in drug use among individuals with BPD and SUD who completed group DBT in comparison to TAU [96]. Further, BS groups were more effective than TS groups in improving psychosocial functioning and decreasing substance use [93]. Finally, CBT was more effective than DRM in reducing alcohol use, enhancing psychological functioning and improving social and family functioning compared to DRM and TAU among individuals dually diagnosed with SUD and a personality disorder [18].

### Factors associated with treatment efficacy

#### Gender and treatment efficacy

Five of the studies included in the present review examined whether treatment was differentially effective for men and women. Although Jarrell and Ridgely’s [93] evaluation of group BS, group TS and individual case management for individuals with SUD and co-occurring Axis I disorders did not examine whether group treatment types were differentially effective for men and women, data indicated that women—regardless of treatment group—reported higher role functioning (i.e.., independent living, work productivity, as well as immediate and extended social relationships), increased psychiatric symptomatology (depression, mania, drug use, alcohol use) across the follow-up periods compared to men.

#### Race and ethnicity and treatment efficacy

Among the studies included in the present review, only three examined whether treatment efficacy varied as a function of race and ethnicity. A secondary examination of the efficacy of group BS in comparison to group TS and individual case management [93] suggested that outcomes in each group treatment among ethnic and racial minority clients were equivalent to White participants during the 6-month follow [94]. The initial evaluation indicated that—regardless of group treatment type—racial/ethnic minority participants reported lower scores in personal well-being, lower life satisfaction (i.e., satisfaction with living), worse role functioning (i.e., independent living, work productivity, immediate and extended social relationships) over the follow-up periods compared to White participants [93].

## Conclusions

In general, participants in group treatment for drug use disorders exhibit more improvement on typical measures of outcome (e.g., abstinence & use rates, objective measures, urinalysis) when compared to standard care without group [18, 109] and those who refuse or drop out of treatment [110]. Specifically, CBT and CM appear to be more effective at reducing cocaine use than TAU groups. CM is effective in increasing periods of abstinence among users of methamphetamine. Both relapse prevention and social support group therapy were effective for marijuana use although relapse prevention was more helpful for men than for women. Brief MI and relapse prevention were both effective at reducing marijuana use. CBT and CBT-related treatments (including the matrix model) when added to pharmacotherapy were more effective for opioid use disorder than pharmacotherapy alone. Effective treatments for Mixed SUD include group CBT, CM, and women’s recovery group. Longer relapse prevention periods appear to be more helpful in reducing mixed SUD. Behavioral skills and behavioral skills plus contingency management helped decreased psychiatric symptoms and drug use behaviors. Psychoeducation groups alone, a commonly used intervention, were not effective at addressing SUD and co-occurring psychiatric problems. Additionally, it is important to note that there is potential for risk of bias in the studies included across four domains: participants, predictors, outcome, and analysis [111]. The current study did not comprehensively assess for risk of bias and this is a study limitation. Future research could assess for risk of bias by following the guidelines suggested by the Cochrane Handbook [112].

The current literature offers a wide variety of group treatments with varying goals and based on varying formal change theories. Overall, studies that reported between-group effect size (*n* = 7) reported small to medium effect sizes potentially suggesting differences were moderate but of potential theoretical interest. Of those seven studies, only two studies reported large effect sizes (both comparing an active treatment to a delayed treatment/untreated condition). In order to better characterize magnitude of intervention effects, future studies should report effect sizes and their confidence intervals [113, 114]. Moreover, groups based on cognitive-behavioral theory [35], motivational enhancement theory [43], stages of change theory [115], 12-step theory [41] and psychoeducational group models [116] have all been the subject of recent studies. Steps of treatment have also been used to classify groups for acutely ill individuals with SUD versus middle stage (recovering) or after care groups, with the latter mainly focusing on relapse prevention. Group therapy is provided – at least as an augment to multimodal interventions – in most of the outpatient and inpatient programs in English speaking and European countries [17, 117]. Therefore, continued efforts to implement and scale up group-based treatments for SUD known to be effective are needed. CM appears to be effective at addressing various drug use problems and further research should evaluate whether it would also be useful for marijuana use.

## Future Research Questions

- Studies of other group treatments for SUD that use rigorous, interview-based diagnosis, use control groups, randomly assign participants to condition, report the ethnic and racial composition of the sample, are adequately powered, implement a treatment manual, and compare outcomes to individual treatment as well are necessary.
- Little is known regarding the possible mediators and moderators of treatment outcome in group interventions for SUD

## Key Learning Objectives

- Group treatment approaches are widely utilized and are often less costly to implement than individual treatments, currently we know very little whether one group approach is superior to another in the treatment of SUD.
- Group treatment approaches seem to be more effective at improving positive outcomes (e.g., abstinence, use rates, objective measures, urinalysis) when compared to standard care without group [18, 109], and those who refuse and drop out of treatment
- More thorough randomized controlled trials of group SUD treatments are needed [110].

## Data Availability

Not applicable. The present study does not include original data. However, the authors of the study have listed all articles reviewed in this study in the reference section.

## Abbreviations

12SG: Twelve Step Facilitation Group Therapy
AD: Alcohol Dependence
AIDS: Acquired Immunodeficiency Syndrome
ASI: Addiction Severity Index
ASPD: Antisocial Personality Disorder
AP: Abbreviated Program
BS: Behavioral Skills
BPD: Borderline Personality Disorder
CBT: Cognitive Behavioral Therapy
CD: Cocaine Dependence
CIDI: Composite Diagnostic Interview Schedule
CM: Contingency Management
CRA: Community Reinforcement Approach
CS: Coping Skills
CT: Cognitive Therapy
DBT: Dialectical Behavioral Therapy
DT: Day Treatment
DC: Drug Counseling
DIS: Diagnostic Interview Schedule
DSM: Diagnostic and Statistical Manual
DRM: Disease and Recovery Model
DTCL: Delayed to Control
EBP: Evidence-Based Practice
EBT: Evidence-Based Treatment Practice
EN: Enhanced Group Condition
ETP: Enhanced Treatment Program
FT: Family Therapy
GDC: Group Drug Counseling
HIV: Human Immunodeficiency Virus
HHRP: HIV Harm Reduction
HR: Harm Reduction
IGT: Intensive Group Therapy
IT: Individual Therapy
MI: Motivational Interviewing
MM: Matrix Model
MMT: Methadone Maintenance Therapy
NIDA: National Institute of Drug Abuse
PET: Psychoeducational Therapy Group
PPWC: Pre-Post with Comparison Group (matched or otherwise)
PTSD: Post Traumatic Stress Disorder
RAWC: Random Assignment with Control
RP: Relapse Prevention
RT: Recovery Training
RAAT: Random Assignment to Active Treatment
RPMG: Relational Psychotherapy Mothers’ Group
SCID: Structured Clinical Interview for Diagnosis
SS: Social Support
SGT: Standard Group Therapy
SUD: Substance Use Disorder
SS: Seeking Safety
STND: Standard Group Counseling
STP: Standard Treatment Program
TAU: Treatment as Usual
TEL: Phone Monitoring and Counseling, with Support Group
TPC: Therapeutic Community Program
TS: Twelve Step
WRG: Women’s Recovery Group
